# LC–MS-based lipidomic analysis of liver tissue sample from spontaneously hypertensive rats treated with extract hawthorn fruits

**DOI:** 10.3389/fphar.2022.963280

**Published:** 2022-08-09

**Authors:** Luping Sun, Bingqing Chi, Mingfeng Xia, Zhen Ma, Hongbin Zhang, Haiqiang Jiang, Fang Zhang, Zhenhua Tian

**Affiliations:** ^1^ College of Pharmacy, Shandong University of Traditional Chinese Medicine, Jinan, China; ^2^ Innovative Institute of Chinese Medicine and Pharmacy, Shandong University of Traditional Chinese Medicine, Jinan, China; ^3^ Experimental Center, Shandong University of Traditional Chinese Medicine, Jinan, China

**Keywords:** hawthorn, spontaneously hypertensive, untargeted metabolomics, lipidomics, liver tissue

## Abstract

At present, many experiments provide support for the cardiovascular protective effect of hawthorn (*Crataegus oxyacantha*) flower, leaf and fruit extracts. The aim of this study was to investigate the intervention mechanism of hawthorn fruit extract on spontaneously hypertensive rats (SHR) and its effect on their lipid metabolic pattern. After SHR was intervened by hawthorn extract (1.08 g/kg/d) for 6 weeks, the blood pressure and liver histopathology of rats were evaluated. An UHPLC-Q Extractive metabolomics approach was used to collect information on rat liver lipid metabolites, combined with multivariate data analysis to identify significantly different substances and potential biomarkers through mass spectrometry and database searches. Histomorphology of the liver was partially restored in the hawthorn-treated group. Hawthorn extract interferes with sphingolipid metabolism, glycerophospholipid metabolism and glycerolipids metabolism, improving partially disturbed metabolic pathways. This study showed that hawthorn could partially restore liver histomorphology and has anti-hypertensive effect by regulating lipid metabolism.

## Introduction

Cardiovascular and cerebrovascular diseases (CVDs) caused by disorders of the heart and blood vessels, are the most morbid, disabling and mortal diseases by far in China, which include coronary heart disease (heart attack), cerebrovascular disease (stroke), elevated blood pressure (hypertension), etc., (Rastogi et al., 2016). Hypertension is one of the main risk factors for CVDs and can increase the risk of heart, brain, kidney and other diseases (Verma et al., 2021). Hypertension is defined as persistent systolic blood pressure (SBP) ( ≥140 mmHg) and diastolic BP (DBP) ( ≥90 mmHg), which is mainly of two types: Primary or essential hypertension (90%–95%) and Secondary hypertension (5%–10%) (Cloud et al., 2019). Furthermore, it is the major cause of premature death worldwide, and its complex pathogenesis is the focus of CVDs research. The most direct and effective treatment for hypertension is to reduce blood pressure through various means (Mills et al., 2020). In recent decades, angiotensin converting enzyme inhibitors (ACE inhibitors), angiotensin receptors blockers (ARBs), direct vasodilators, calcium channel blockers, ganglion blockers, and thiazide type of diuretics are the main clinical drugs in the treatment of hypertension (Goit and Yang, 2019). At present, the clinical treatment focuses on the combined use of a variety of antihypertensive drugs. Nebivolo/valsartan combination is effective and well-tolerated to obtain excellent antihypertensive effect (Wang and Sander, 2021). However, it has been reported that the risk of adverse reactions of antihypertensive drugs in subjects is high, such as stroke, coronary heart disease and cardiovascular diseases (Gronewold et al., 2019).

Most herbs from different regions have multiple anti-cardiovascular effects (Rastogi et al., 2016). Hawthorn (*Crataegus oxyacantha*) is the ripe fruit of a plant in the Rosaceae family and is used worldwide as a food and medicine. In particular, a large number of experimental studies have proved that hawthorn has the effects of anti-hypertension, protecting vascular endothelial function, anti-arrhythmia, regulating lipid metabolism, lowering blood lipids, inhibiting platelet function, decreasing oxidative stress, and reducing inflammatory response (Chang et al., 2005; Zhao et al., 2017). The hawthorn flower and leaf extract possess antioxidant effect and blood-thinning properties (Rababa’h et al., 2020). The rich polyphenolic components of hawthorn peel and pulp could differentially modulate dyslipidemia, inflammation, oxidative stress, and alleviate liver injury in mice, which is the main reason for its excellent antioxidant and free radical scavenging abilities (Han et al., 2016; Zhao et al., 2017). The mediated cardiogenic, vasodilatory and antioxidant effects of flavonoid bioactive compounds in hawthorn extracts making them beneficial in the prevention of pulmonary hypertension syndrome and heart disease (Ahmadipour et al., 2020). Flavonoids and oligomeric proanthocyanidins play a major role in the protection and treatment of various CDVs (Rigelsky and Sweet, 2002).

Hypertension is a clinical syndrome characterized by varying degrees of lipid metabolism disorder (Xie, et al., 2019). Chinese medicine considers hypertension be-longing to dizziness, headache, stroke, liver wind. It occurs the pathogenesis induces wind, fire, phlegm, empty, stasis. These five aspects extremely are closest with the liver relations (Wang et al., 2014). Liver is an essential organ of lipid metabolism (Trefts et al., 2017) and serves as the main site of carbohydrate and lipid biosynthesis in the body (Jones, 2016). Lipids are important small biomolecules and could maintain lipid metabolic homeostasis. Moreover, it is an essential element of the mammalian life form (Nguyen et al., 2008a). Overexpression of lipids can lead to excessive accumulation of fat in hepatocytes, increase triglycerides and low-density lipoprotein cholesterol levels, decrease high-density lipoprotein cholesterol levels and insulin resistance, eventually leading to elevated blood lipids and aggravating the occurrence and progress of spontaneous hypertension (Nguyen et al., 2008b). Thus, lipid disorders can indeed aggravate the occurrence and progress of spontaneous hypertension. Lipidomics analysis can reveal changes in lipid metabolism and related regulatory mechanisms in response to internal and external stimuli (Duan and Xie, 2020). Lipidomics may be an useful tool for identifying biomarkers of visceral fat and liver fat content (Boone et al., 2019). Liquid chromatography-mass spectrometry (LC-MS) has been widely used for lipidomics analysis of various samples with the advantage of high sensitivity and high resolution to determine more metabolic phenotypes of polar metabolites by untargeted and targeted analysis (Gika et al., 2019; Guo et al., 2021).

In this study, we investigated the effects of hawthorn fruit extract on the metabolism of spontaneously hypertensive rats using untargeted LC-MS liposome analysis. This study provides new insights into the mechanism of hawthorn treatment in spontaneously hypertensive rats.

## Materials and methods

### Chemicals and reagents

HPLC-grade methanol, acetonitrile, formic acid water, and 2-propanol were purchased from Tedia Co., Inc. (Fairfield, OH, United States). All aqueous solutions were prepared using ultrapure Milli-Q water (EMD Millipore, Billerica, MA, United States).

### Preparation of the extract of hawthorn fruit

Hawthorn fruit (No. 20121602) were purchased from Shandong Shunshengtang Prepared Slices of Chinese Crude Drugs Co., Ltd. (Heze, Shandong, China), which were subjected to chemical analysis according to the Chinese Pharmacopoeia and identified as satisfactory. Ultrasonic extraction was performed with hawthorn-70% ethanol (1:9, m/v) for 1 h, and repeated for 2 times. After filtration, the extract was concentrated by decompression and dried. When administered in animal experiments, 3.0 g/ml extract was prepared by dissolving the extract in physiological saline. Hawthorn extract solution were stored at 4°C until used in the animal study.

### Animal experiment

12 Wistar-Kyoto rats (WKYs, aged 12 weeks) and 36 male Spontaneously Hypertensive Rats (SHRs, aged 8 weeks), each weighing 190 ± 10 g, were procured from the Vital River Laboratory Animal Technology Co., Ltd. (Beijing, China, Permit No. SCXK Lu 20170022). All experimental procedures were performed in accordance with the guidelines of the guidelines of the National Research Council of China and were approved by the Animal Ethics Committee of the Affiliated Hospital of Shandong University of Traditional Chinese Medicine (SDUTCM20210721002).

The 12 WKYs were defined as the control group (C), and the 24 SHRs were randomly divided into model group (M, *n* = 12) and hawthorn treated group (S, *n* = 12). All rats were kept under controlled environmental conditions (temperature of approximately 23°C ± 3°C, humidity of 55% ± 15% and a 12 h light/dark cycle). All rats were acclimatized to the laboratory 1 week before the experiments. They were allowed free access to food and water throughout the study. Drug administration in rats calculated by human dose (9–12 g/d) in the Chinese Pharmacopoeia and the dose of hawthorn extract in group S was 1.08 g/kg/d (Xu et al., 2002; Chen, 2006). The Groups of C and M were intragastric with equal amount of normal saline. All animals were intragastric once daily for 6 weeks.

### Detection of systolic blood pressure and diastolic BP

Both of SBP and DBP were measured for each rat in each group before the experiment and every week (1–6 weeks respectively) conducted by the noninvasive blood pressure apparatus (Dotop Biotech Co., Ltd., BP-2000).

### Collection and preparation of samples

At the end of the experiment, all animals were fasted for 12 h, the rats were anesthetized with sodium pentobarbital, and blood was collected from the abdominal aorta. At the same time, the fresh tissue of the right lobe of the liver was rapidly removed and the surface blood was washed with pre-cooled normal saline. Part of the liver sample was immersed in 4% paraformaldehyde and fixed for subsequent detection, and the remaining part was placed in a centrifuge tube and stored in a—80°C refrigerator for lipid analysis.

Quality control (QC) samples were used to rule out the possibility that significant differences in liver lipid metabolites analyzed by LC-MS were primarily caused by instrument drift, and thus to measure instrument stability. QC samples were obtained by taking 20 μL of each liver tissue homogenate and processing the mixture in the same manner as the other samples. 5 consecutive QC samples were performed to equilibrate the system before the actual liver tissue samples were tested, and then a QC sample was inserted into every 5 samples (Yang et al., 2020). The obtained QC sample data matrix was subjected to principal component analysis (PCA) to further monitor the system stability.

### ELISA kit test

100 mg of liver tissue stored at −80°C was added with 900 μL precooled isopropyl alcohol for rapid homogenization, centrifuged at 4°C for 12,000 r/min for 15 min, and then NO (Cat.#m1058803-2) and TNF-α (Cat.#m1002859-2) levels in liver samples were determined by ELISA (Shanghai Enzyme-linked Biotechnology Co., Ltd., Shanghai, China) according to manufacturer’s instructions. The concentrations of NO and TNF-α in liver samples were calculated according to the calibration curve.

### Hematoxylin and eosin staining and oil red staining

The same part of the liver tissues were fixed with 10% formalin and embedded in paraffin blocks, which were further sectioned at 5 μm and stained hematoxylin and eosin (H&E). 10% formalin-fixed fresh liver tissue was embedded with OCT embedding gel and sectioned for 5 μm using a freeze sectioning machine, then, stained with oil red O and hematoxylin. Finally, the sections were then photographed with Axiophot 2 upright microscope (Axio Imager. A2).

### UHPLC-Q extractive analysis

Liver tissue (100 mg) and water (900 μL) were homogenized for 1 min, then centrifuged at 12,000 rpm for 15 min at 4°C to remove water-soluble impurities. Then, the supernatant was discarded and dichloro-methanol (2:1, v/v) was added to the lower tissue pellet and vortexed for 5 min. After centrifugation at 12,000 rpm for 15 min at 4°C, the bottom layer was carefully aspirated and dried with high-purity nitrogen. Finally, it was resolved with 100 μL isopropanol-acetonitrile-water (2:1:1, v/v/v), collected using a 0.22 μm microporous membrane and filtered with 3 μL into a column for UHPLC-Q Extractive analysis (Furse et al., 2020).

The data analysis was performed using a UHPLC-Q Extractive (Thermo Fisher Scientific) equipped with a Aegla-Hlao C_18_ column (2.1 mm × 100 mm, 2.7 μm). The mobile phases were 0.05% formic acid in acetonitrile-water (4:6, v/v) solution (A) and 0.05% formic acid in acetonitrile-isopropanol (1:9, v/v) solution (B), the gradient program is as follows: 0–3 min, 2%–45% B; 3–10 min, 45%–65% B; 10–25 min, 65%–85% B; 25–25.2 min, 2% B. The injection volume was 3 μL, and the flow rate was 0.3 ml/min. The column was maintained at 45°C.

An electrospray ionization ion (ESI) source was used for mass detection in positive and negative mode, and the drying gas temperature and drying gas flow rate were set to 350°C and 10 ml/min, respectively. The capillary voltage was set at 3,500 V, the atomization gas pressure was set at 35 psi, the fragmentation voltage was 140 V and the skimmer voltage was 60 V. Data were collected in centroid mode from 100 to 1,000 m*/z*. Normalized Collision Energy (NCE) was 30, 50, and 70 eV.

### Data processing and multivariate analysis

Peak alignment and extraction were performed using R (R Project V3.2.2, The University of Auckland, New Zealand) (Zhang et al., 2016), and the original data were converted and normalized. The processed normalized data were imported into SIMCA-P (V14.1, MKS Data Analytics Solutions Umea, Sweden) for multivariate analysis, including principal component analysis (PCA) and partial least squares discriminant analysis (PLS-DA) for pattern recognition. The purpose of PCA is to eliminate outlier samples by similarity between the groups, and the aims of PLS-DA is to identify the significantly changed metabolites of plasma samples in different groups (Dong et al., 2020). According to a score plot of PLS-DA, we selected the variable importance prediction (VIP) of the projected first principal component greater than 1 to screen the differential metabolites for further analysis (Yang et al., 2017). Then, the VIP >1 data of SHR group and normal group were imported into Mass Profiler Professional (V12.6.1, Agilent Technologies, United States ) software for *t*-test. Values with *p* values less than 0.05 and fold changes greater than 2 were retained as potential biomarkers (Tian et al., 2020).

The Human Metabolomics Database online (HMDB, www.hmdb.ca) was used for metabolite identification. According to the relevant literature (Alseekh et al., 2021), the accurate molecular weight and tandem mass spectrometry results, the mass spectrometry information in the library was matched, and the types of differential metabolites were further identified. At the same time, the analysis of compounds was also carried out with reference to the Kyoto encyclopedia of genes and genomes (KEGG, www.kegg.com) (Guma et al., 2016). Other bioinformatics tools for path mapping and network visualization, including MetaboAnalyst 5.0 (dev.metaboanalyst.ca), are also used in this study. Finally, the metabolic pathways were drawn.

### Statistical analysis

Data from ELISA assays were processed with SPSS 26.0 (SPSS Inc., United States ) statistical software. Data were expressed as mean ± standard deviation (SD) for processing independent sample *t*-test and one-way analysis of variance (ANOVA). *p* < 0.05 indicated that the difference was statistically significant.

## Results

### Hawthorn extract reduces blood pressure in spontaneously hypertensive rats

**FIGURE 1:**
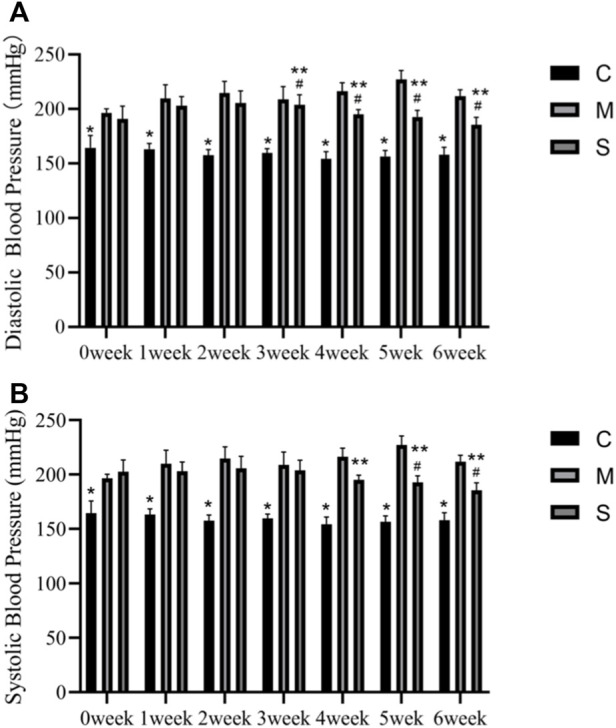
**(A)** Diastolic blood pressure in the normal control (C), SHR model (M) and hawthorn treated (S) rats. **(B)** Systolic blood pressure in the normal control (C), SHR model (M) and hawthorn treated (S) rats. *p < 0.05 vs. model group, ^#^ *p* < 0.05 vs. 0 weeks, ^##^ *p* < 0.05 vs. SHR.

Figure 1 shows the comparative results of diastolic and systolic blood pressure in normal control (C), SHR model (M) and hawthorn treated (S) rats during 6 weeks. SBP and DBP were significantly higher in both group M and group S without pharmacological intervention (0 weeks) than in group C. Compared with the M group, the S group showed a significant decrease in SBP at 4, 5, and 6 weeks (*p* < 0.05) and a decrease in DBP from the 3rd week (*p* < 0.05). Compared to the pre-intervention S group (0 weeks), the SBP decreased at 5 and 6 weeks (*p* < 0.05) and DBP decreased significantly at 3, 4, 5, and 6 weeks (*p* < 0.05) (Figure 1).

### Hawthorn extract promotes NO and inhibites TNF-α production

**FIGURE 2:**
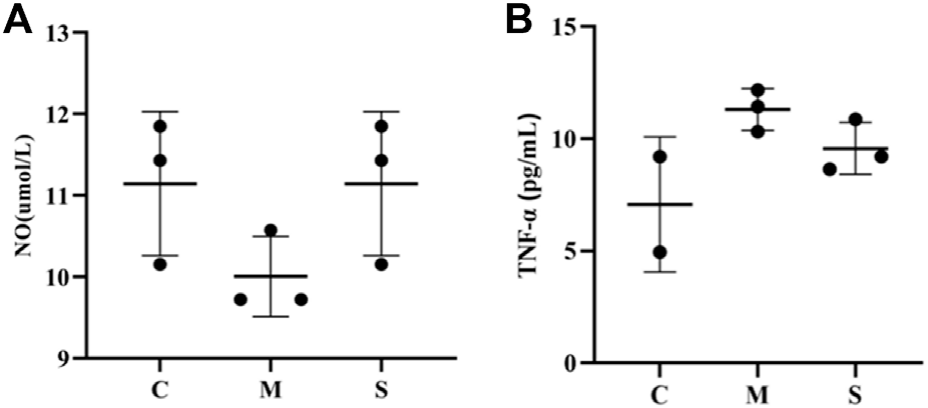
**(A)**The NO level in liver tissues of in normal group (C), model group (M) and hawthorn group (S). **(B)**The TNF-α levels in liver tissues of in normal group (C), model group (M) and hawthorn group (S).

NO and TNF-α indexes were measured in rat liver tissue homogenates by ELISA. Compared with the normal control group (C), NO was significantly decreased in the model group (M), while TNF-α levels were significantly increased (*p* < 0.01). After administration of the treatment, NO levels in the S group were improved and significantly increased (*p* < 0.01), while the decrease in TNF-α levels was not significant (Figure 2). It proved that hawthorn could achieve therapeutic effects by elevating NO and decreasing TNF-α.

### Hawthorn extract improves liver histological abnormality

**FIGURE 3:**
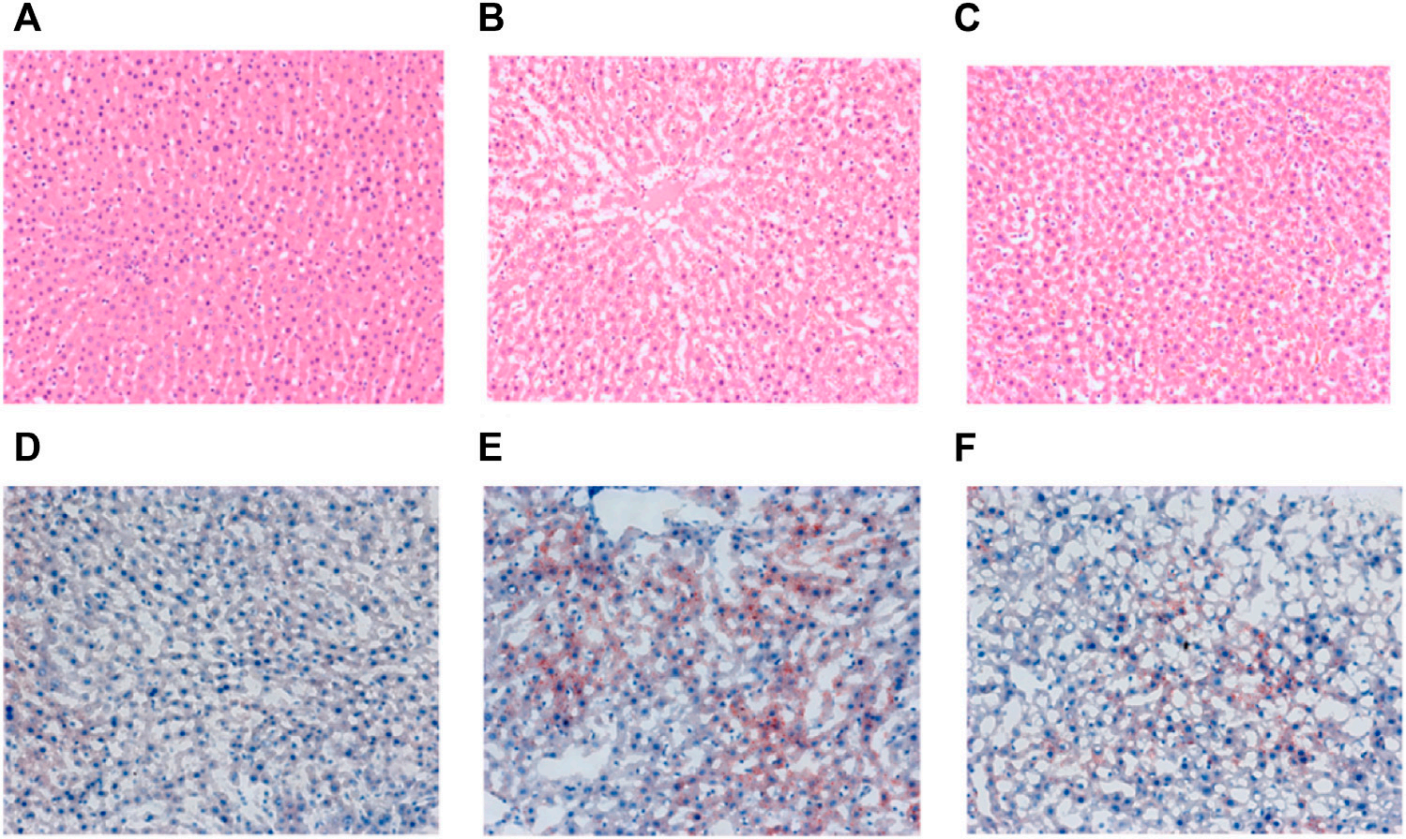
Representative H&E staining (magnification, × 200) of liver sections from the C **(A)**, M **(B)** and S **(C)** groups and oil red staining (magnification, × 200) of liver sections from the C **(D)**, M **(E)** and S **(F)** groups at the end of 6th week.

At the end of the 6th week of the experiment, the liver tissue of the healthy control (C) group was clear, and no histological abnormality was found in the liver section, while in the SHR model (M) group, liver cells showed obvious fatty change and small necrotic area (Figure 3). Compared with the M group, the steatosis of the hepatic cells in the S groups was reduced, and the fat content of the hepatic cells was significantly reduced.

The oil red O staining showed that a large number of red lipid droplets appeared in the hepatocytes of SHR in group M compared with group C, and the area of oil red staining was significantly increased, indicating that their intracellular lipid content was significantly higher than that of group C. In contrast, the number of red intracellular lipid droplets was significantly reduced in the S group treated with hawthorn compared to the M group, implying a significant reduction in lipid content (Figure 3). The staining results proved that hawthorn can significantly improve liver pathology, reduce the degeneration of SHR hepatocytes, and lower the lipid content in the liver, making it a practical and effective drug for improving cardiovascular disease (Dong et al., 2020).

### Multivariate data analysis and pattern recognition

**FIGURE 4:**
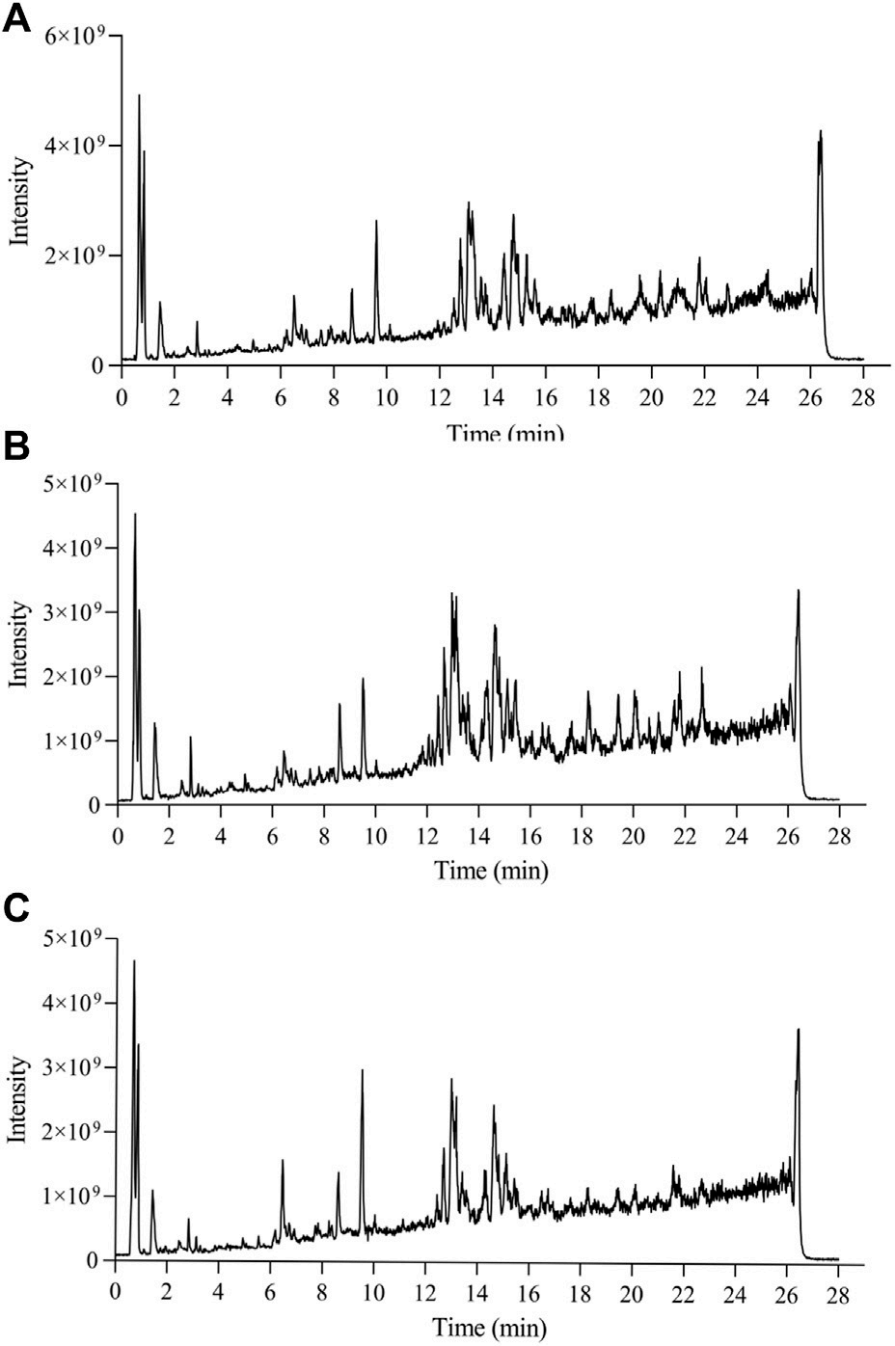
Total ion chromatograms of WKY **(A)**, SHR **(B)** Hawthorn treated **(C)** in positive ion mode obtained from UPLC-QE-MS analysis.

Representative total ion chromatograms of the liver samples from the QC groups in the positive (A) and negative (B) ESI mode was shown in Supplementary Figure S1 and UHPLC-Q Extractive analysis Total ion Chromatograms (TIC) of liver tissue samples from each experimental group were shown (Figure 4; Supplementary Figures S2, S3). To confirm the good stability and applicability of the metabolomics platform, we extracted the retention time and peak area of each of the five representative ions in QC samples in positive and negative ion mode using the analytical method in the literature, and calculated the RSD value <5% for the corresponding intensity of each ion (Supplementary Tables S1, S2), indicating that the instrument was in good condition, data quality was reliable. To further perform method validation to obtain the deviation variation of QC samples, the stability of QC was evaluated by principal component analysis, and the results showed that the 12 QC samples in both positive and negative ion mode were within the region of 2SD and 95% confidence interval (Supplementary Figure S4).

**FIGURE 5:**
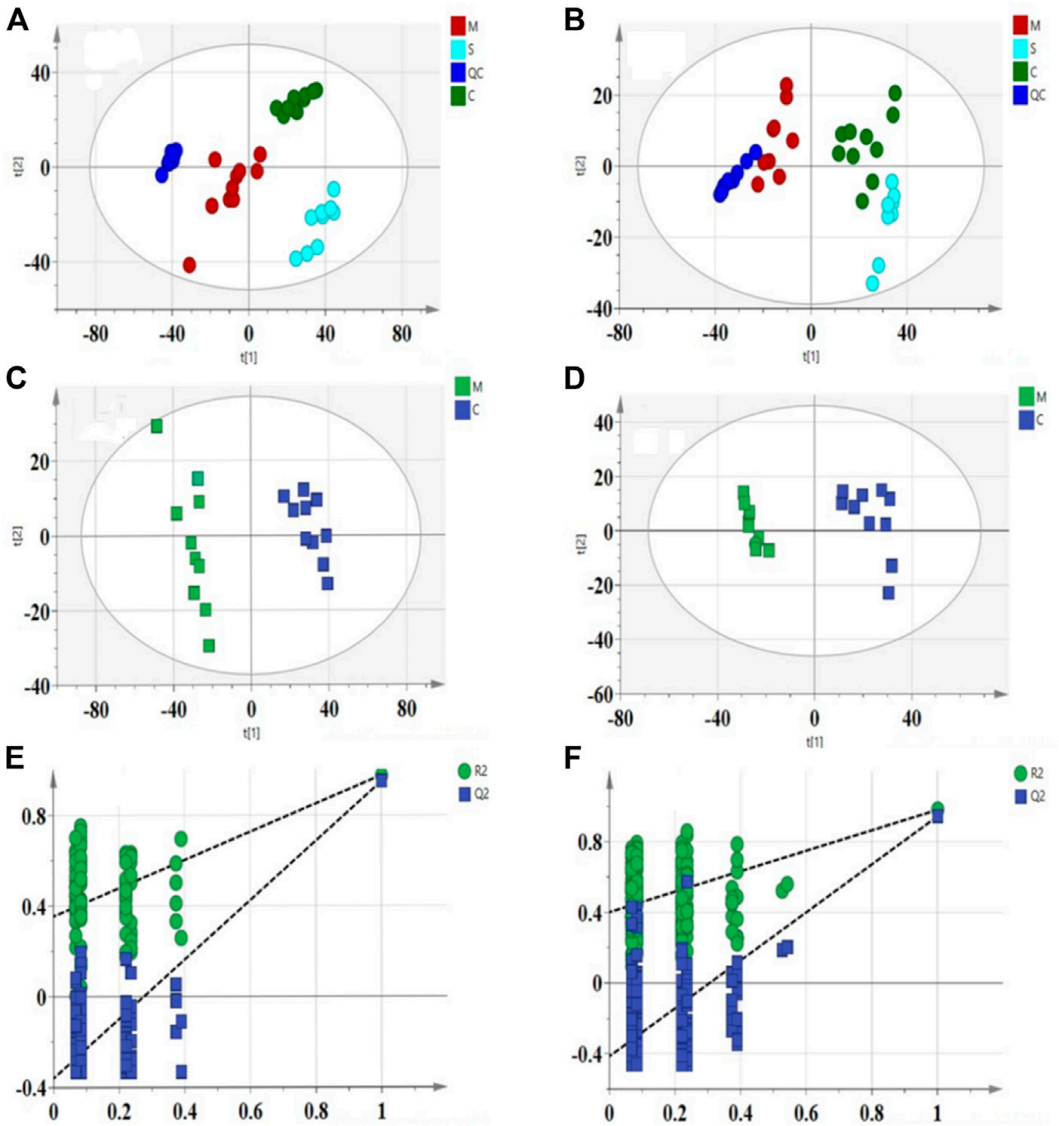
PCA score scatter plots for liver samples from 3 groups in the positive **(A)**: R^2^X = 0.872, Q^2^ = 0.785) and negative **(B)**: R^2^X = 0.745, Q^2^ = 0.661) ESI mode. OPLS-DA score scatter plots for C and M groups in the positive **(C)**: R^2^X = 0.626, R^2^Y = 0.998, Q^2^ = 0.98) and negative **(D)**: R^2^X = 0.814, R^2^Y = 0.9958, Q^2^ = 0.966) ESI mode. Permutation test of lipid species OPLS-DA model in positive mode **(E)**: *R* ^2^ = 0.354, Q^2^ = −0.359) and negative mode **(F)**: *R* ^2^ = 0.47, Q^2^ = −0.447).

Principal component analysis (PCA) is an unsupervised pattern recognition method in multivariate statistical analysis, which can obtain classification information between different samples and clearly show the two-dimensional spatial distribution relationships between different groups, and the results are shown as score plots. In this study, the PCA model was used to assess the metabolomic differences between the 3 groups and all 3 groups were significantly separated in both positive and negative modes, as shown in Figures 5A,B). The results showing the PCA score plots for lipids in positive and negative ionization mode. The positive mode (R^2^X = 0.872, Q^2^ = 0.785) and the negative mode (R^2^X = 0.745, Q^2^ = 0.661) indicate that the developed PCA model has good predictive and explanatory power. The QC samples were found to be significantly clustered and concentrated within the 95% confidence interval in the PCA score scatter plot, providing the necessary conditions for a large-scale metabolomics study.

To further elucidate the differences in lipid metabolism patterns among the groups, this experiment used partial least squares discriminant analysis (PLS-DA), a more mature supervised projection method, to analyze the data of each group and obtain PLS-DA scatter plots (Figures 5C,D), from which it can be seen that the three groups of samples appeared clearly spatially classified without crossover or overlap, and the PLS-DA models of R^2^X, R^2^Y, and Q^2^ are within the specified ranges of 0.626, 0.998, 0.98 (positive mode) and 0.814, 0.996, 0.966 (negative mode), respectively, implying that the experimentally established model is valid. The study established the alignment test as an internal validation method to demonstrate the validity of the OPLS-DA model. The 200 permutation tests for the positive ion model (*R* ^2^ = 0.354, Q^2^ = −0.359) and the negative ion model (*R* ^2^ = 0.47, Q^2^ =−0.447) (Figures 5E,F) showed that the intercept of Q^2^ on the vertical axis was seen to be negative. All the established OPLS-DA models are sreliable and robust with no over-fitting and the model validation is valid.

### Analysis and identification of potential biomarkers and lipid metabolic pathways in hypertension

The variable importance prediction (VIP) values generated by the PLS-DA model were used to filter variance variables. The larger the VIP value of a variable, the greater its contribution to the group. Potential endogenous components with VIP >1 were screened and further independent samples *t*-test and fold change (FC) analysis were performed to select variables with *p* < 0.05 and FC > 2. In conclusion, we considered variables that satisfied both VIP >1, *p* < 0.05 and FC > 2 as potential biomarkers. Based on the above conditions, 31 differential variables were identified as potential biomarkers, including 13 in the positive ion mode and 18 in the negative ion mode, and the differences and trends of these biomarkers were summarized in Table 1.

**TABLE 1:** Identification of ions and their alteration trend in positive and negative mode.

No	tR (min)	m/z	Formula	Identification	Fragments	Change trend^#^		Pathway
						Control^#^	Hawthorn^#^	
1	6.56	691.5278	C45H70O5	DG (22:6/20:3/0:0)	M + H	↓	↓	Glycerolipids
2	6.72	697.4424	C41H65O8P	PA (18:4/20:4)	M-H20-H	↑	↑	Glycerophospholipids
3	9.52	678.6843	C44H87NO3	Cer(18:1/26:0)	M + H	↓	↓	Sphingolipids
4	11.81	937.5278	C49H81N2O11 PS	PC(18:4)	M + H	↓	↓	Glycerophospholipids
5	11.89	863.5125	C44H82O13P2	PGP (20:1/18:2)	M + H-H2O	↓	↓	Glycerophospholipids
6	12.21	889.5266	C45H81N2O11 PS	PC(14:0)	M + H	↓	↓	NC
7	12.35	699.5023	C39H73O9P	PA (18:0/18:1)	M + H-H2O	↓	↓	NC
8	12.36	698.4993	C39H72NO7P	PE (16:0/18:3)	M + H	↓	↓	Glycerophospholipids
9	12.49	728.5277	C40H76NO9P	PE (18:3/16:0)	M + H-H2O	↓	↓	Glycerophospholipids
10	12.83	939.5446	C49H83N2O11 PS	PC(18:3)	M + H	↓	↓	NC
11	12.99	816.5313	C46H78NO10P	PS(20:3/20:3)	M-H20-H	↓	↓	Glycerophospholipids
12	13.19	794.5302	C44H80NO10P	PS(20:2/18:1)	M-H20-H	↓	↓	Glycerophospholipids
13	13.57	784.5012	C42H76NO10P	PS(20:3/16:0)	M-H	↓	↓	Glycerophospholipids
14	14.13	770.5297	C42H80NO10P	PS(18:1/18:0)	M-H20-H	↓	↓	Glycerophospholipids
15	14.31	868.5643	C50H82NO10P	PS(22:6/22:2)	M-H20-H	↓	↓	Glycerophospholipids
16	14.36	794.5477	C44H80NO10P	PS(20:3/18:0)	M-H20-H	↑	↑	Glycerophospholipids
17	14.78	859.5980	C47H87O11P	PA (24:0)	M + H	↓	↓	NC
18	15.91	808.5985	C46H84NO8P	PE-NMe(22:4/18:0)	M-H	↑	↑	Glycerophospholipids
19	16.05	676.4808	C36H72NO8P	PE (16:0/15:0)	M-H	↑	↑	Glycerophospholipids
20	16.09	902.6041	C46H80O13P2	PGP (22:6/18:0)	M-H	↑	↑	Glycerophospholipids
21	17.61	849.6478	C57H88O6	TG (20:5/14:1/20:5)	M-H20-H	↑	↑	Glycerolipids
22	17.68	823.6308	C55H86O6	TG (14:1/20:5/18:4)	M-H20-H	↑	↑	Glycerolipids
23	18.25	735.5132	C39H75O10P	PA (18:1/18:0)	M + H	↓	↓	NC
24	18.34	629.5506	C41H76O5	DG (18:1/0:0/20:1)	M-H20-H	↑	↑	Glycerolipids
25	18.58	836.6542	C49H94NO8P	PE-NMe2 (18:1/24:1)	M-H20-H	↑	↑	Glycerophospholipids
26	19.46	927.6815	C63H94O6	TG (18:4/22:5/20:5)	M-H20-H	↑	↑	Glycerolipids
27	19.47	853.6671	C57H92O6	TG (14:0/20:5/20:4)	M-H20-H	↑	↑	Glycerolipids
28	20.06	873.7097	C57H94O6	TG (14:0/20:4/20:4)	M-H	↓	↓	Glycerolipids
29	22.84	839.5599	C47H83O10P	PA (i-24:0)	M + H	↑	↑	NC
30	23.99	625.5183	C41H70O5	DG (38:5)	M + H-H2O	↓	↓	Glycerolipids
31	24.29	901.5058	C46H80O13P2	PGP (18:1/22:5)	M-H	↓	↓	Glycerophospholipids

Model^#^: Trends of the model group compared with the control group of metabolites. Hawthorn^#:^ Trends of the treatment group compared with the model group of metabolites. NC: not classified.

**FIGURE 6:**
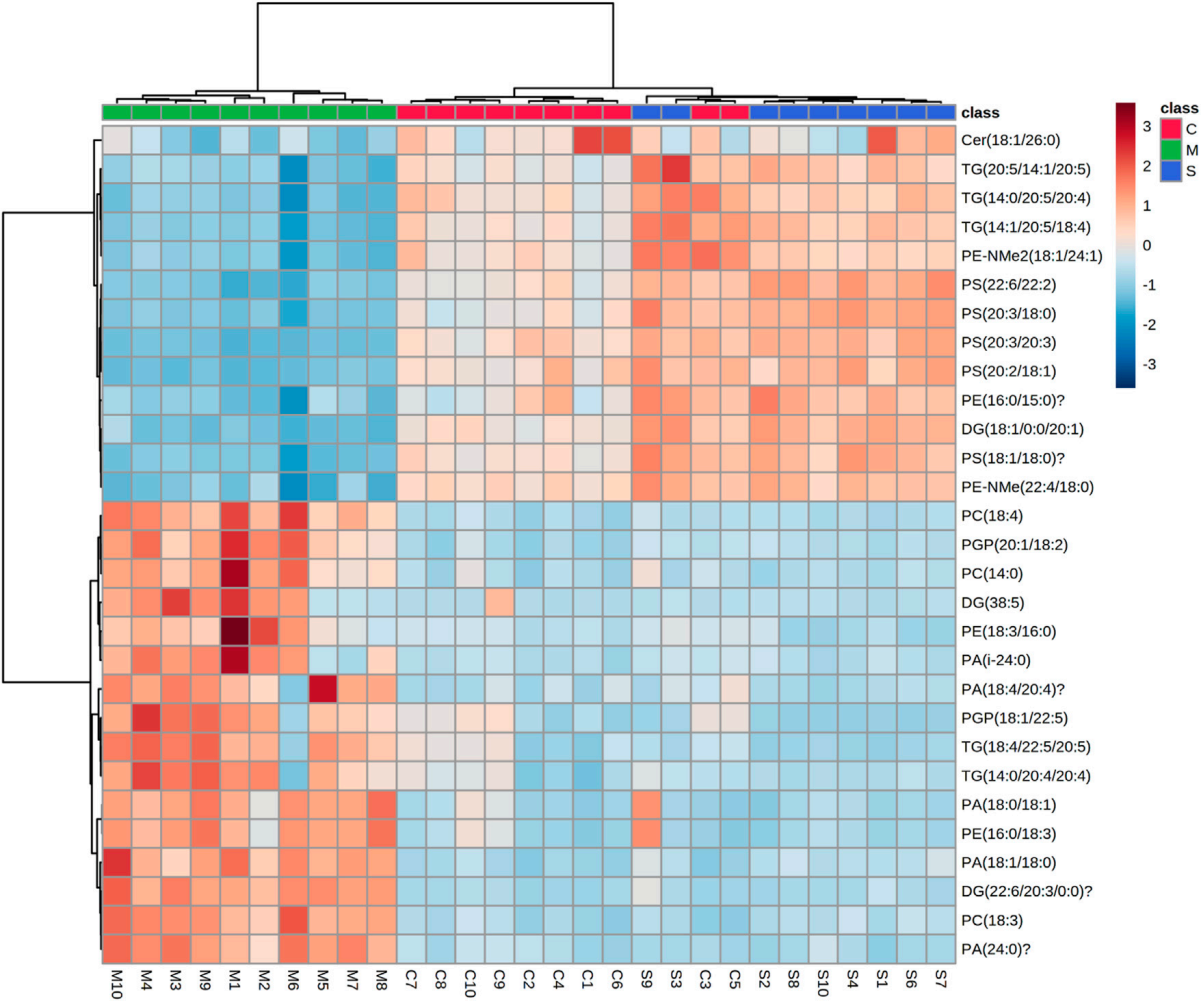
Heat map of the 31 differential lipid metabolites between the control (C1-C10) group, model (M1-M10) group and S (S1-S4 and S6-S10) group. Rows: samples; columns: metabolites. Dark red plots indicated upregulated metabolites and dark blue plots indicated downregulated metabolites in rats.

We constructed a clustering heatmap using MetaboAnalyst 5.0 (dev.metaboanalyst.ca), which is able to characterize the relative content of each component to analyze the identified lipid data (Figure 6). The heatmap shows the differences in the content of the identified lipids, with dark blue indicating lower levels and dark red indicating higher levels. Based on the information in the graph, it can be observed that the color change in the hawthorn treatment group (S) has converged to the level of the normal group (C). For example, the levels of ceramides (Cer, 18:1/26:0), p-glycoprotein (PGP, 20:1/18:2; 18:1/22:5), phosphatidic acid (PA, 18:0/18:1; 24:0), phosphatidylcholine (PC, 18:4; 14:0; 18:3), phosphatidylethanolamine (PE, 16:0/18:3; 18:3/16:0), phosphatidylserine (PS, 20:3/20:3; 20:2/18:1; 20:3/16:0; 18:1/18:0; 22:6/22:2), diglycerides (DG, 22:6/20:3/0:0; 38:5) and triglycerides (TG, 14:0/20:4/20:4) were significantly higher in the model group than in the control group, but the metabolite levels returned to the control group after treatment with hawthorn, which indicates that the metabolite levels were regulated and the abnormalities of lipid metabolism were improved to varying degrees with the intervention of hawthorn, suggesting that hawthorn has a regulatory effect on lipid disorders in the liver of SHR.

**FIGURE 7:**
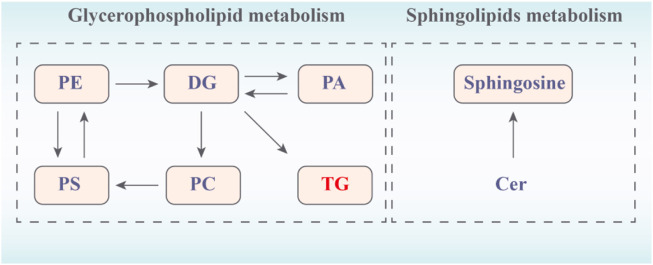
Network of changes in potential biomarkers of SHR regulated by hawthorn (red: upregulated, blue: downregulated).

Then, to explore the signaling pathways that may be affected by hawthorn, we performed metabolic pathway analysis of the identified lipid differential metabolites. Figure 7 showed that sphingolipid metabolism and glycerophospholipid metabolism. The results showed that 3 metabolic pathways: sphingolipid metabolism, glycerophospholipid metabolism and glycerolipids metabolism were all disturbed, implying that these metabolic pathways were significantly associated with the effect of hawthorn in treating SHR.

## Discussion

The effect of hawthorn extract on SHR was studied. Under experimental conditions, the regulation effect of hawthorn on improving lipid metabolism is moderate and positive. 3 metabolic pathways were identified by lipidomics. Thus, hawthorn can regulate liver lipidomics through sheath lipid metabolism, glycerin phospholipid metabolism and glycerin metabolism, thereby regulating lipid content and exerting its therapeutic effect on hypertension. A total of 31 lipid types closely related to hypertension were identified in this study, mainly produced in the plasma of hepatocytes, including fatty acids, glycerolipids (including TG, DG), and glycerophospholipids (including PC, PE, PS, and PA) (Alves-Bezerra and Cohen, 2017). Hepatocytes are the major hepatic parenchymal cells that control hepatic biochemical and metabolic functions and play a key role in lipid metabolism (Gong et al., 2017). These lipid overexpressions can result in excessive accumulation of fat in hepatocytes, and ultimately lead to elevated blood lipids, which are the main risk factors for hypertension (Graessler et al., 2009).

Glycerophospholipids and sphingolipids are common in all tissues, and sphingolipids are the most important polar lipids in biology (Castro-Gómez et al., 2015). Based on the nature of their polar groups, glycerophospholipids are broadly classified as PC, PE, PS, phosphatidylinositol (PI), PA and cardiolipin (CL) (Castro-Gómez et al., 2015; Wang et al., 2020). PC and PE are the two most abundant phospholipids in all mammalian cell membranes (Wang et al., 2020). It has been shown that inhibition of PC synthesis impairs the secretion of very low density lipoproteins (LDL) and that abnormal changes in PC or PE levels in various tissues lead to metabolic disorders and are associated with disease progression, such as hypertension, atherosclerosis, insulin resistance and obesity (van der Veen et al., 2017). PE could use different substrates and enzymes to synthesize PS through two metabolic pathways. In the presence of the same ER enzyme, the reaction is reversible, and PS can in turn regenerate PE and serine (Castro-Gómez et al., 2015).

Sphingolipids consist of PC or PE, and mainly include Cer, sphingomyelin (SM), and glycosphingolipids (GSLs), which are involved in various indispensable metabolic, neural and intracellular signaling processes (Castro-Gómez et al., 2005; Wang et al., 2020). Sphingolipids are broken down by sphingomyelinase to produce Cer and PC which accelerate the formation of DG by phosphatidylcholine. DG is the final product of the lipid signaling pathway, and plays a role in regulating protein kinase C activity and calcium release with second messenger inositol 1,4,5-triphosphate (Au et al., 2017). TG could be lipolyzed to large amounts of free fatty acids (FFA). FFA contribute to the development of hypertension by causing a sustained accumulation of DG and TG in the liver and muscle, leading to endothelial diastolic dysfunction, inactivating NO oxidation and activating some serine/threonine kinases (Kulkarni et al., 2013), which may induce hypertension lipotoxicity (Kulkarni et al., 2013; Hu et al., 2010). In fact, compared with WKY, the results of this study showed that increased Cer (18:1/26:0), PC (18:4; 14:0; 18:3) and PE (16:0/18:3; 18:3/16:0) in SHR accelerated the release of PS (20:3/20:3; 20:2/18:1; 20:3/16:0; 18:1/18:0; 22:6/22:2), DG (22:6/20:3/0:0; 38:5), resulting in increased TG (14:0/20:4/20:4). However, these changes were apparently reversed after hawthorn treatment. Therefore, hawthorn can play a therapeutic role in hypertension by regulating Cer, PC, PE, PS, DG and TG. It is worth noting that compared with WKY, the NO content in SHR was significantly reduced, indicating that hypertension was accompanied by endothelial dysfunction. However, after the intervention of hawthorn, the NO content was higher than that of SHR, which indicated that hawthorn had a certain improvement effect on endothelial dysfunction and played a role in the treatment of hypertension.

In summary, this study revealed the overall changes in the hawthorn extract treatment of hypertension by lipidomics technology, a total of 31 biomarkers and 3 pathways were identigfied. The results showed that after hawthorn treatment, the blood pressure level, liver lipid accumulation and lipid metabolism disorder in SHR were positively alleviated. Additionally, the vascular endothelial damage was improved and inflammatory factors inhibited in liver. In general, hawthorn could antagonize hypertension by regulating specific lipid molecules through lipidomics.

## Data Availability

The original contributions presented in the study are included in the article/Supplementary Material, further inquiries can be directed to the corresponding authors.
