# Supplementary material for: LC–MS-based lipidomic analysis of liver tissue sample from spontaneously hypertensive rats treated with extract hawthorn fruits
Source: Front Pharmacol. 2022 Aug 9;13:963280. doi: 10.3389/fphar.2022.963280 (PMC9395718; doi:10.3389/fphar.2022.963280)
Supplement: Supplementary file 1 [file DataSheet1.pdf]

Supplementary Materials

LC–MS-Based Lipidomic Analysis of Liver Tissue Sample from Spontaneously Hypertensive Rats Treated with Extract Hawthorn Fruits

Luping Sun[1#], Bingqing Chi [1], Mingfeng Xia[2], Zhen Ma[2], Hongbin Zhang[1], Haiqing Jiang[2]*, Fang Zhang[1]* , Zhenhua Tian[3]*

[1] College of Pharmacy, Shandong University of Traditional Chinese Medicine, Jinan 250000, China
[2] Innovative Institute of Chinese Medicine and Pharmacy, Shandong University of Traditional Chinese Medicine, Jinan 250000, China
[3]Experimental Center, Shandong University of Traditional Chinese Medicine, Jinan 250000, China

*Corresponding author:
Zhenhua Tian, E-mail: tianzhenhuatina@163.com; Experimental Center, Shandong University of Traditional Chinese Medicine, Jinan 250000, China. Tel: +86-0531-8962014
Fang Zhang, E-mail: zfang_819@163.com, College of Pharmacy, Shandong University of Traditional Chinese Medicine, Jinan 250000, China.
Haiqing Jiang, E-mail: jhq12723@163.com, Innovative Institute of Chinese Medicine and Pharmacy, Shandong University of Traditional Chinese Medicine, Jinan 250000, China. Tel: 86-0531-89628192

Contents

Figure S1 Representative total ion chromatograms of the liver samples from the QC groups in the positive (A) and negative (B) ESI mode.
Figure S2 Total ion chromatograms of WKY (**C**), SHR (**M**) Hawthorn treated (**S**) in positive ion mode obtained from *UPLC-QE-MS* analysis.
Figure S3 Total ion chromatograms of WKY (**C**), SHR (**M**) hawthorn treated (**S**) in negative ion mode obtained from *UPLC-QE-MS* analysis.
Figure S4 PCA line score plots of different injections of QC sample. X-axis represented the run order of QC sample; Y-axis represented standard deviation (A, C) and Hotelling's T2 range (B, D), separately. (A, B) for positive ESI mode; (C, D) for negative ESI mode.
Figure S5 Fingerprints of hawthorn (No. 20121602) within one week (R: control; S1: Monday; S2: Wednesday; S5: Friday; S7: Sunday).
Table S1 Relative standard deviation (RSD) values of 10 ion signals of QC in the positive ion mode
Table S2 Relative standard deviation (RSD) values of 10 ion signals in negative ion modes

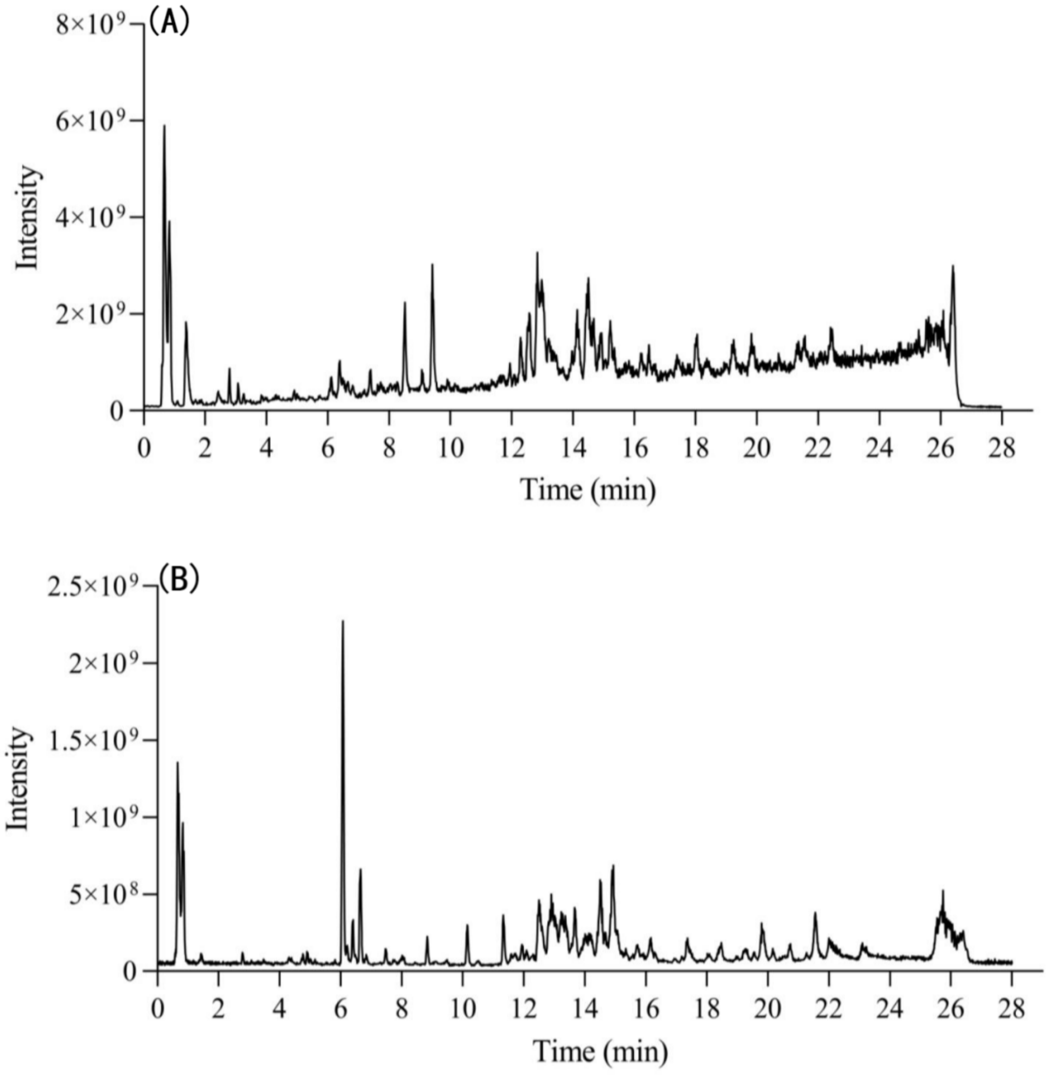

Figure S1. Representative total ion chromatograms of the liver samples from the QC groups in the positive (**A**) and negative (**B**) ESI mode.

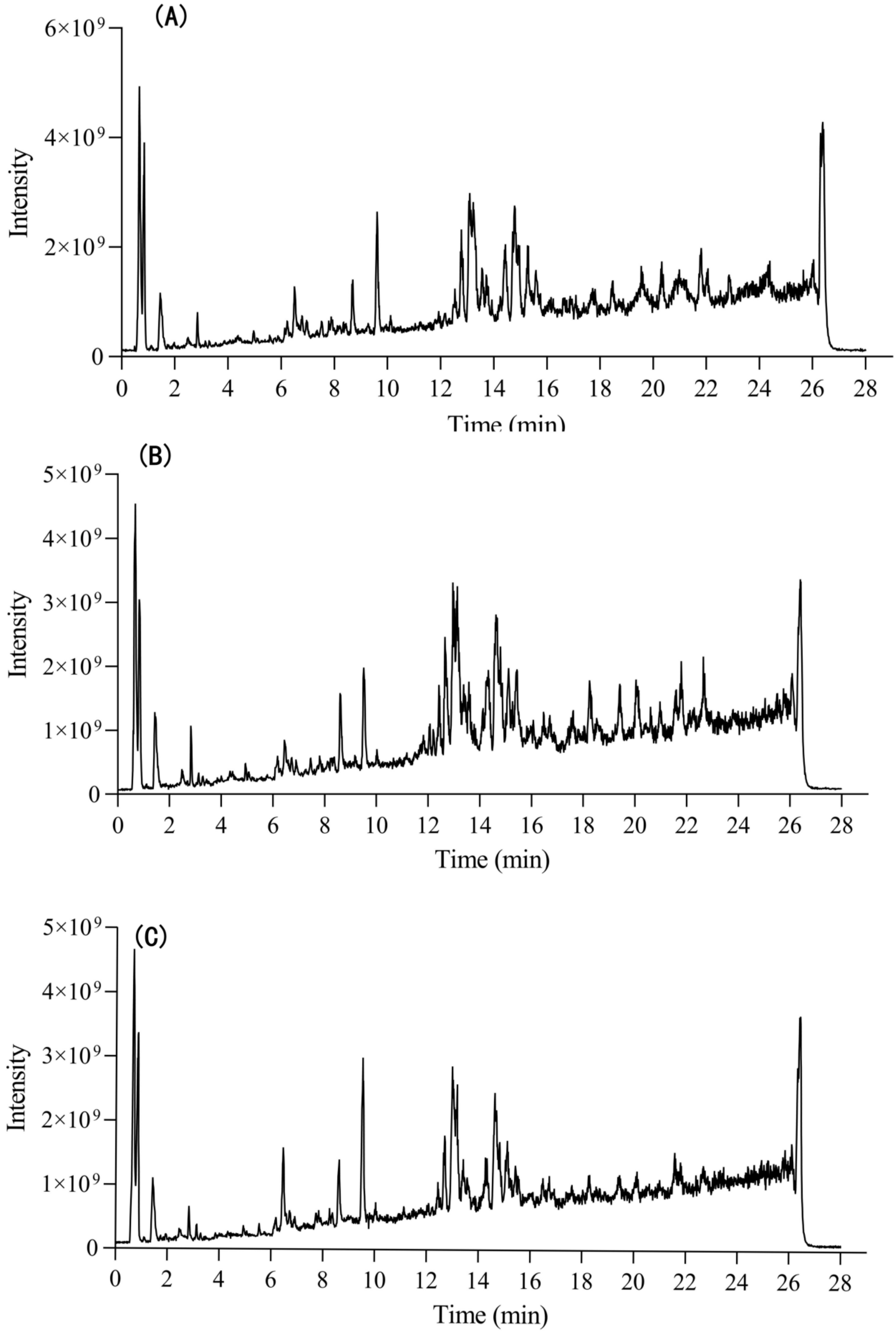

Figure S2. Total ion chromatograms of WKY (**C**), SHR (**M**) Hawthorn treated (**S**) in positive ion mode obtained from UPLC-QE-MS analysis.

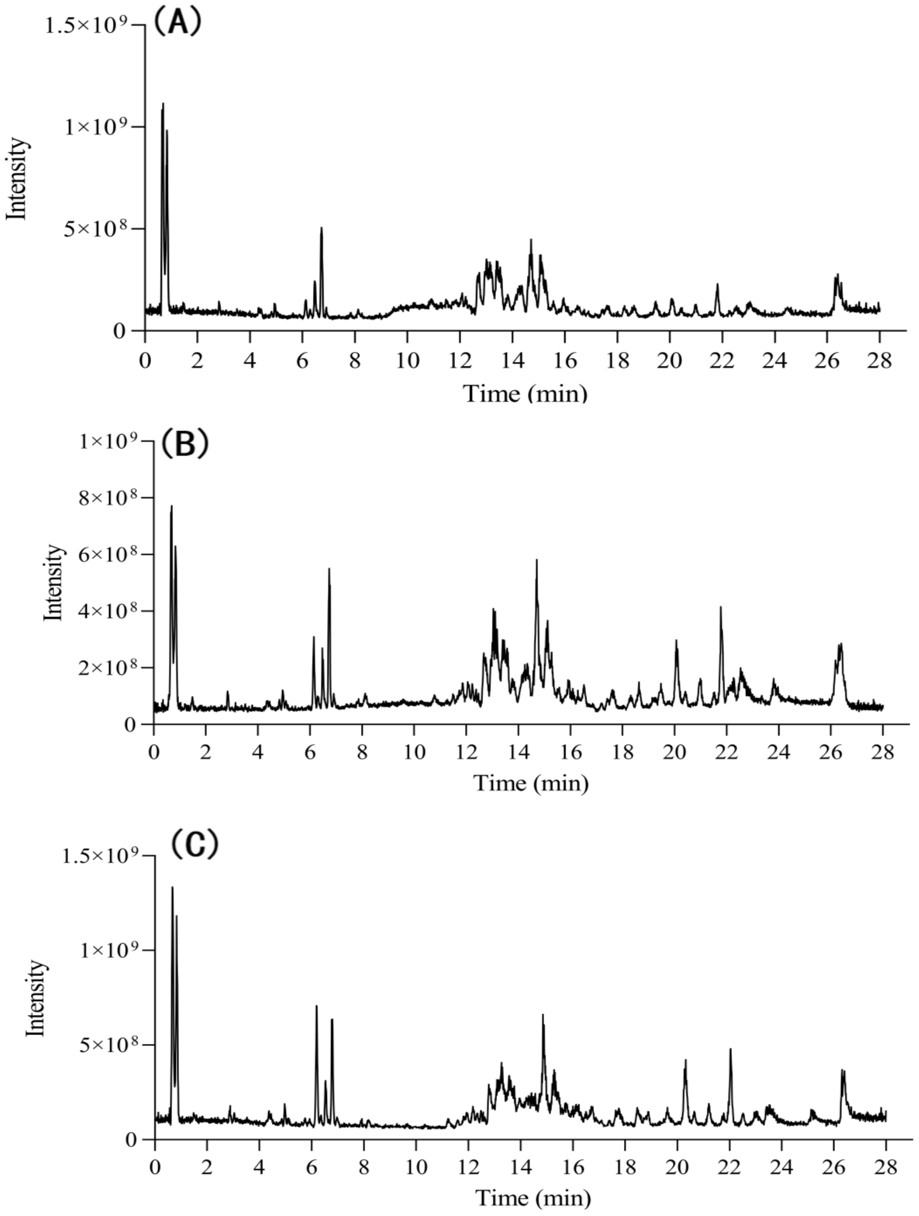

Figure S3. Total ion chromatograms of WKY (**A**), SHR (**B**) hawthorn treated (**C**) in negative ion mode obtained from UPLC-QE-MS analysis.

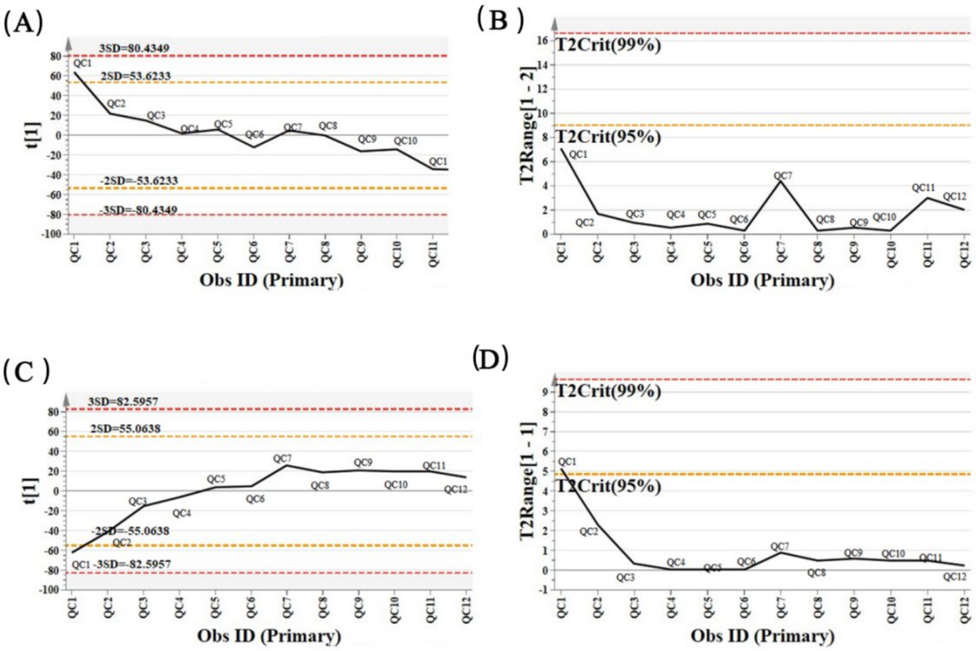

Figure S4. PCA line score plots of different injections of QC sample. X-axis represented the run order of QC sample; Y-axis represented standard deviation (A, C) and Hotelling's T2 range (B, D), separately. (A, B) for positive ESI mode; (C, D) for negative ESI mode.

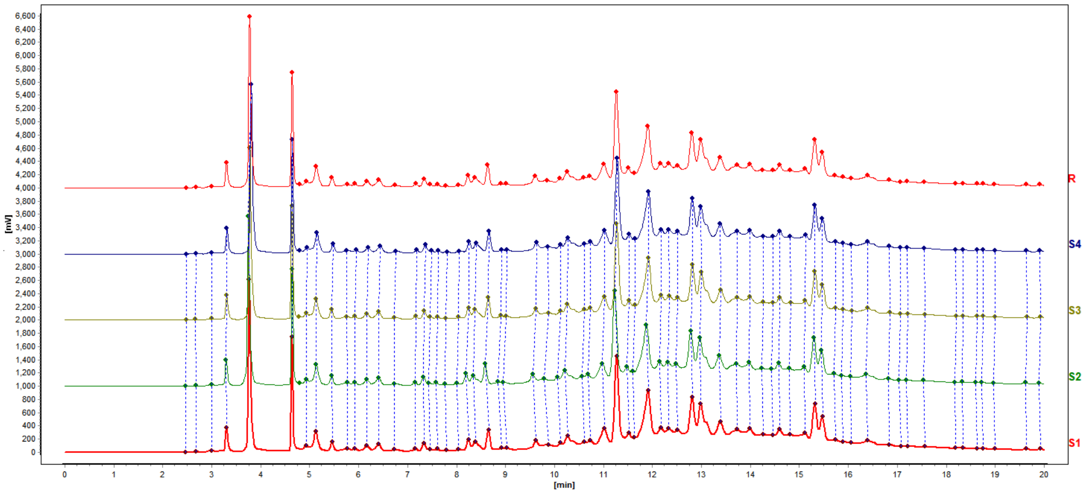

Figure S5. Fingerprints of hawthorn (No. 20121602) within one week (R: control; S1: Monday; S2: Wednesday; S5: Friday; S7: Sunday).

Table S1 Relative standard deviation (RSD) values of 10 ion signals of QC in the positive ion mode

**Positive mode** (m/z)	**RT** (min)	**Instrument repeatability** RSD (%, intensity, n=6)
281.9937	0.58	9.62
193.1623	0.60	8.60
332.3229	3.13	2.28
625.3135	6.12	7.61
293.2830	7.91	4.54
451.2653	8.61	5.48
672.4746	10.90	3.56
979.6212	13.34	6.71
433.8247	17.66	7.17
911.2246	20.50	4.44

Table S2 Relative standard deviation (RSD) values of 10 ion signals in negative ion modes

**Positive mode** (m/z)	$t_{\mathbf{med}}$ (min)	**Instrument repeatability** RSD (%, n=12)
433.8247	1.45	7.39
193.1623	2.93	5.31

625.3135	4.83	7.99
332.3229	6.14	6.35
281.9937	12.47	7.74
451.2653	13.19	4.00
451.2653	16.08	8.41
979.6212	17.61	6.90
293.2830	20.08	5.49
672.4746	23.80	4.82
